# The relationship between serum transglutaminase-2 levels and the severity of chronic spontaneous urticaria

**DOI:** 10.1007/s10238-024-01422-z

**Published:** 2024-07-22

**Authors:** Omneya M. Zeyada, Zeinab A. Ashour, Omar A. Lotfy, Mayada M. Mahmoud

**Affiliations:** 1 Department of Internal Medicine/Allergy and Clinical Immunology, Faculty of Medicine, Ain Shams University, 11591 Abbasia, Cairo Egypt; 2 Department of Internal Medicine, El Zaitoun Specialized Hospital, Cairo, Egypt

**Keywords:** Chronic spontaneous urticaria, Severity, TG2, UAS

## Abstract

Chronic spontaneous urticaria (CSU) is an immunological disease that is depicted by high prevalence and eminent burden for patients and society that is attributable to the arbitrary nature of symptoms and inconsistent tools for assessment of activity and severity. Transglutaminase-2 (TG2) is a posttranslational enzyme that is pervasively expressed in many cells and tissue types including mast cells. It has various biological functions, and its role in allergic disorders has been highlighted and delineated through several postulated mechanisms. This case–control study aimed at determining the relationship between serum levels TG2 and severity of CSU. To the best of our knowledge, this is the first study in Egypt to determine the relationship between serum TG2 and severity of CSU. We enrolled 60 adult patients with confirmed diagnosis of CSU. According to urticaria activity score (UAS), patients were categorized into three groups [20 with mild disease; UAS = 0, 20 with moderate disease; UAS = 1–3, 20 with severe disease; UAS = 4–6]. Another 20 healthy individuals (age and gender matched) served as a control group. All patients were subjected to detailed medical history, clinical examination, complete blood count with differential, serum total IgE, CRP, ESR, TSH, ANA, liver and renal function tests. Serum level of TG2 was done by quantitative ELISA for all enrolled patients and controls. Serum TG2 is significantly higher in patients group compared to control group (*P* value < 0.001). Serum TG2 levels were significantly higher in patients with severe disease compared to patients with moderate or mild disease. This is illustrated by the significant positive correlation between serum TG2 and UAS (*r* 0.814 and *P* value 0.000). Moreover, serum TG2 accurately classified CSU patients into mild, moderate and severe subgroups: as regards differentiation between mild and moderate cases (sensitivity 70%, specificity 80%, PPV 77.8, NPV 72.7) and as for the differentiation between moderate and severe cases (sensitivity 95%, specificity 90%, PPV 90.5, NPV 94.7). Serum TG2 may have a pivotal role as a marker of severity in patients with CSU.

## Introduction

Chronic spontaneous urticaria (CSU) is a highly prevalent immunological disease affecting about 1% of the world population. It is characterized by skin manifestations in the form of recurrent wheals which are vigorously itchy. The wheals may be associated with angioedema in about 40–50% of patients. Symptoms of CSU are usually unprovoked with almost no role of external factors, nevertheless, concomitant infection and elevated stress levels are significant aggravators of the disease activity. The burden of the disease is paramount for patients and society with robust effects on health-related quality of life. These effects stem from the prolonged course that can last for years, severity of symptoms, unpredictable nature and associated co-morbidities [1].

The pathogenesis of CSU is an exemplification of internal inflammatory process of the skin. The fundamental initiating step is the activation of dermal mast cells (MCs) for which two autoimmune hypotheses have been postulated. The first mechanism is mast cell degranulation due to reaction of IgE with an autoallergen in the skin (Type I autoallergy). The second mechanism is mediated by IgG antibodies directed against IgE or the extracelluar α subunit of the high affinity IgE receptor (FceRI) (type IIb autoimmunity). IgG autoantibodies can activate the complement pathway which augments MCs degranulation [2]. However, the processes underlying the disruption of the immune system leading to autoantibody formation are not well identified [3]. The activated mast cells at the site of wheals release vasoactive substances and inflammatory mediators such as histamine, leukotrienes, platelet-activating factor, cytokines and chemokines which result in endothelial cell activation with consequent vasodilatation, increased vascular permeability and perivenular infiltration of inflammatory cells. This cascade prompts skin inflammation, edema and pruritis [4].

In CSU, the disease usually persists for 1–5 years and this duration is prone to increase in direct proportion with the severity of the condition [1]. Among the many challenges in the management of patients with CSU, a principal feature is the arbitrary pattern of recurring symptoms. Moreover, the lack of a definitive curative therapy renders the situation more complex. The contemporary lines of treatment claim to focus on the control of disease activity and prevention of recurrence of symptoms. This reflects on the significance of the decision of choosing the correct treatment and the point of time to switch treatments [5]. At the current time, assessment of the activity and control of the disease is exclusively clinical involving medical history and physical examination reinforced by validated yet subjective and retrospective measures of patient-reported outcomes questionnaires like Urticaria Activity Score (UAS), Chronic Urticaria Quality of Life (CUQ2oL) and Urticaria Control Test (UCT). Recent reports from literature have advocated the implementation of blood parameters as indicators of activity and potential prognostic biomarkers in CSU [6]. To date, the most propitious candidates are D-dimer, CRP and IL-6 demonstrating significantly higher levels in severe cases of CSU. However, the indeterminate conclusions about their specificity to CSU and consistent association with the disease contradict the key characteristics that are considered pivotal for a biomarker [7]. Hence, the recognition, validation and implementation of an objective consistent biomarker in CSU could pave the way for the administration of a personalized approach to the evaluation and prediction of the disease course and outcomes as well as tailored treatment plans [8].

Transglutaminase 2 (TG2) is a posttranslational enzyme that is pervasively expressed in many cells and tissue types including mast cells. It acts through modification of proteins to exert its various biological functions. These include maintenance of cell membrane integrity, modulation of signal transduction, formation of skin barrier and assembly of extracellular matrix. The salient part that TG2 takes in important biological processes raises the likelihood of its contribution in the evolution of autoimmune, inflammatory and degenerative diseases, like, rheumatoid arthritis, celiac disease and pulmonary fibrosis [9]. Its role in allergic disorders has also been highlighted through the activation of phospholipase A2 (PLA2) and nuclear factor (NFekB) which prompt increase in eicosanoids mediating allergic inflammation. TG2 induces expression of IL-33 and downstream molecules leading to differentiation of TH2 and initiation of allergic TH2 response [10]. Moreover, recent observations have identified increase in IgE production through TG2 expressed and activated in mast cells [11]. Therefore, it can be presumed that TG2 activity may be implicated in the pathogenesis of CSU and in particular its increased activity and serum level in correlation to severity of the condition.

This study aimed at determining the relationship between serum levels of TG2 and severity of CSU.

## Methods

### Study design and study subjects

We conducted a comparative case–control study over a period of 6 months. We enrolled 60 adult patients with confirmed diagnosis of CSU selected by systematic randomization from patients attending the Allergy & Immunology clinic at Ain Shams University Hospitals. Another 20 healthy individuals (age and gender matched) served as a control group. Written and verbal consent was taken from all participants.

Considering the results of a previous study by Bae et al. [15] that showed the mean level of TG2 was higher among patients with CSU than in non-allergic normal controls, the sample size was calculated to be, 20 mild, 20 moderate and 20 severe CSU patients and 20 non-allergic normal controls, at a confidence level of 95% and power of study 80%. These figures were approved by the Research Ethics Committee at the Faculty of Medicine, Ain Shams University (FMASU REC).

### Inclusion and exclusion criteria

CSU was diagnosed according to according to the standard criteria in EAACI/GA^2^LEN/EDF/WAO guidelines [12]. Inclusion criteria were adult patients (≥ 18 years old) clinically diagnosed with CSU. Exclusion criteria included patients with inducible urticaria, patients with symptoms or signs that may render the diagnosis of CSU uncertain (eg, fever, arthralgia, acute lymphadenopathy), patients with mixed connective tissue diseases, diabetes mellitus, hypertension, renal diseases, liver diseases, autoimmune disorders, malignancies, inflammatory bowel disease and celiac disease. No patients received systemic steroids or immunotherapy within 1 month of enrollment.

Patients included in the study were subjected to detailed medical history including allergic history and clinical examination. CSU severity and activity were assessed according to the EAACI/GA2LEN/EDF1 guidelines. Venous blood samples were withdrawn from CSU patients for CBC, CRP, ESR, TSH, liver function tests and renal function tests, ANA, total serum IgE and from patients and healthy controls for serum level of TG2.

### Assessment of chronic spontaneous urticaria activity and severity

According to the EAACI/GA2LEN/EDF1 guidelines, the severity of CSU was measured using UAS which is an integrated scoring system based on the evaluation of the fundamental signs and symptoms of urticaria (wheals and pruritis) as follows: Wheals score [0 = none, 1 = mild wheals ie. < 20/24 h, 2 = moderate ie.20–50/24 h, 3 = intense ie. > 50 /24 h or large confluent areas of wheals]. As regards pruritis score [0 = none, 1 = mild ie. present but not annoying, 2 = moderate ie. annoying but no interference with daily activities or sleep, 3 = intense ie. severe pruritis interfering with daily activity or sleep]. The disease activity was rated on a scale from 0–6 as follows: UAS 0 infers controlled urticaria, UAS 1–3 infers low activity, UAS 4–6 infers high activity [13]. The values of UAS were estimated on the day before serum TG2 tests were measured for comparison of the disease severity with the test results.

### Laboratory investigations

Data from CSU patients included in the study were collected and recorded concerning the results of CBC, CRP, ESR, TSH, liver function tests (AST and ALT) and renal function tests (Serum Creatinine) and antinuclear antibody (ANA). CBC was done on Sysmex XN 1000, CRP on Copas C (Roche diagnostics), ESR by modified Westergren method, TSH by Electrochemiluminescence immunoassay, AST and ALT according to the IFCC recommendations, serum creatinine by modified Jaffe’s reaction and ANA by indirect immunofluorescence.

### Serum total IgE concentration

Venous blood sample (5 ml) was obtained by venipuncture from CSU patients and collected into a gel Vacutainer tube (Becton Dickinson, Oxford, UK). Blood was allowed to be clotted and serum was separated by centrifugation at 1200 g for 15 min at 25 °C. Separated serum was stored in aliquots at − 20 °C until used for measurement of serum total IgE levels by an enzyme linked immunosorbent assay (ELISA). The estimation of total IgE levels (IU/ml) was done using an ELISA kit (Catalog number: A0141, RIDASCREEN; R-Biopharm, Darmstadt, Germany) and performed according to the manufacturer’s instructions. The normal level of total IgE in adults is less than 100 IU/ml.

### Serum transglutaminase-2 concentration

Venous blood sample (5 ml) was obtained by venipuncture from CSU patients as well as from healthy controls for the assessment of serum TG-2. Serum was separated and allowed to clot for 10–15 min at room temperature. Then centrifugation was done at 2000–3000 RPM for 20 min. The supernatant was then collected without sediment. Samples were stored at − 20 °C until used for the assay procedure. The estimation of serum TG2 was done using commercially available quantitative ELISA Kit supplied by Bioassay Technology Laboratory and performed according to the manufacturer’s instructions. This procedure has a sensitivity of 2.74 ng/L. No repeated freezing nor thawing were allowed.

### Statistical analysis

Descriptive statistics were calculated for all the variables (cases and controls) including continuous variables (reported as mean values and standard deviations or median with interquartile range) and categorical variables (reported as numbers and percentages). The primary exposure variable for all subjects was determined as the quantitative assessment of the level of TG2 in serum. For between-group comparisons, the independent t-test, Mann Whitney test was used for continuous variables and Pearson's *χ*2-test test for nominal variables were appropriate. The relationship between serum level of TG-2 and severity of CSU was summarized based on calculating an odds ratio. Conditional logistic regression models were used to calculate the odds ratio and the corresponding 95% confidence intervals (CIs) with and without adjusting for potential confounding factors. All the statistical analyses were performed using SPSS version 20.0 software, and *P* values less than 0.05 were considered to be statistically significant.

## Results

The current case–control study comprised 60 cases (adult patients with confirmed diagnosis of CSU) and 20 controls (age and gender matched healthy subjects). Further subgrouping of CSU was done based on disease activity and severity into mild, moderate and severe groups (20 patients in each group).

## Basic demographic characteristics and laboratory data of cases

Out of the 60 patients with CSU recruited, females represented 55%. Mean age was 29.77 years (± 7.30). The median value of (UAS) was 2 (IQR = 0, 4). The mean TLC was 6.52 cells/mm^3^ (± 1.79), for neutrophil count 3.31 (± 1.05), lymphocyte count 2.04 (± 0.67) and eosinophil count 0.28 (± 0.10). The median value of C-reactive protein (CRP) was 4 (IQR = 3, 5.5) and for ESR was 18.5 (IQR = 15, 22.5). Mean TSH was 2.43 mIU/mL (± 0.84). Mean total IgE in serum was 225.58 IU/ml (± 58.41). As for the serum level of TG2, the median value was 439.05 ng/L (IQR = 255.4, 628.75).

Table 1 shows that there was no statistically significant difference between control and patients groups regarding gender and age of the studied subjects with *P*-value = 0.696 and 0.597, respectively.

**Table 1:** Comparison between control and patients groups regarding demographic data of the studied subjects

		Control group	Patients group	Test value	*P*-value	Sig
		No. = 20	No. = 60			
Gender	Female	12 (60.0%)	33 (55.0%)	0.152*	0.696	NS
	Male	8 (40.0%)	27 (45.0%)			
Age (yrs)	Mean ± SD	28.85 ± 4.30	29.77 ± 7.30	− 0.530•	0.597	NS
	Range	22–38	19–45			

*P*-value > 0.05: Non significant; *P*-value < 0.05: Significant; *P*-value < 0.01: Highly significant

*: Chi-square test; •: Independent t-test

Table 2 presents the comparison of the results of the laboratory investigations between the three subgroups of CSU patients (mild, moderate and severe). The analysis shows non-significant differences between the three subgroups regarding all illustrated laboratory parameters in Table 4 except for CRP values which show a highly significant difference between the subgroups with the highest values reported in the subgroup with the highest severity.

**Table 2:** Comparison between mild, moderate and severe groups regarding laboratory data

		Mild	Moderate	Severe	Test value	*P*-value	Sig
		No. = 20	No. = 20	No. = 20			
Hemoglobin	Mean ± SD	12.79 ± 1.83	12.58 ± 1.66	13.20 ± 2.02	0.587•	0.559	NS
	Range	9.8–15.6	9–15	9.6–16.1			
Platelets (10^3)	Mean ± SD	287.90 ± 98.58	284.90 ± 77.56	267.25 ± 64.43	0.376•	0.689	NS
	Range	178–526	168–435	167–421			
Total leucocyte count (TLC)	Mean ± SD	6.73 ± 1.73	6.28 ± 1.89	6.57 ± 1.81	0.323•	0.725	NS
	Range	3.7–9.6	3.7–9.9	3.8–9.9			
Neutophils	Mean ± SD	3.43 ± 0.97	3.10 ± 1.13	3.40 ± 1.06	0.584•	0.561	NS
	Range	2–4.75	1.7–5.6	1.8–5.5			
Lymphocytes	Mean ± SD	2.11 ± 0.67	1.98 ± 0.66	2.03 ± 0.70	0.182•	0.834	NS
	Range	1.1–3.5	1.1–3.26	0.8–3.2			
Neut/Lymph ratio	Mean ± SD	1.67 ± 0.35	1.59 ± 0.35	1.72 ± 0.28	0.815•	0.448	NS
	Range	1.1–2.2	1.16–2.55	1.16–2.25			
Esinophils	Mean ± SD	0.30 ± 0.12	0.26 ± 0.08	0.27 ± 0.10	1.216•	0.304	NS
	Range	0.11–0.49	0.12–0.43	0.09–0.46			
CRP	Median (IQR)	2.5 (1–4)	4 (3–5)	5 (3–6)	11.632 ≠	0.003	HS
	Range	1–12	2–9	3–16			
ESR 1st hour	Median (IQR)	11.5 (7.5–14)	10.5 (7.5–13.5)	9 (6–11.5)	2.732 ≠	0.255	NS
	Range	3–26	4–25	2–17			
ESR 2nd hour	Median (IQR)	20 (15.5–25.5)	19 (15.5–23)	16 (14–19)	5.005 ≠	0.082	NS
	Range	11–40	11–39	11–23			
TSH	Mean ± SD	2.62 ± 0.93	2.46 ± 0.90	2.22 ± 0.68	1.162•	0.320	NS
	Range	0.98–4.12	0.96–4.02	0.98–3.41			
Total IgE	Mean ± SD	213.35 ± 52.93	244.60 ± 63.95	218.80 ± 55.82	1.671•	0.197	NS
	Range	145–310	168–400	145–310			

*P*-value > 0.05: Non significant; *P*-value < 0.05: Significant; *P*-value < 0.01: Highly significant

•: One Way ANOVA test;  ≠ : Kruskal–Wallis test

Table 3 presents the comparison between the control and patients (all cases) regarding the serum level of TG2. It is illustrated that there is a highly significant difference between control and cases groups regarding serum TG2.

**Table 3:** Comparison between control and patients groups regarding serum transglutaminase-2

Serum transglutaminase-2	Control group	Patients group	Test value	*P*-value	Sig
	No. = 20	No. = 60			
Median (IQR)	75.34 (63.86–102.04)	439.05 (255.4–628.75)	− 6.333 ≠	< 0.001	HS
Range	53.07–301.2	90.14–1280			

•*P*-value > 0.05: Non significant; *P*-value < 0.05: Significant; *P*-value < 0.01: Highly significant

≠ : Mann–Whitney test

Table 4 presents the comparison of the results of the serum level of TG2 between the control group with the three subgroups of CSU patients showing a highly significant difference and with the analysis of the Post Hoc analysis, it is evident that the most significant differences are illustrated in the comparisons involving the subgroup of patients with moderate and severe disease inferring the relationship between serum TG2 and disease severity.

**Table 4:** Comparison between control, mild, moderate and severe groups regarding serum transglutaminase-2

Serum transglutaminase-2	Control group		Mild		Moderate		Severe		Test value	*P*-value	Sig
	No. = 20		No. = 20		No. = 20		No. = 20				
Median (IQR)	75.34 (63.86–102.04)		237.25 (196.25–285.85)		406.8 (265.4–499.45)		835.3 (605.55–1002.5)		64.946 ≠	0.000	HS
Range	53.07–301.2		90.14–492.4		174.1–801.4		518–1280				
*Post Hoc analysis*											
P1		P2		P3		P4		P5		P6	
< 0.001		< 0.001		< 0.001		0.001		< 0.001		< 0.001	

•*P*-value > 0.05: Non significant; *P*-value < 0.05: Significant; *P*-value < 0.01: Highly significant

≠ : Kruskal–Wallis test

P1: Control Vs mild; P2: Control Vs moderate; P3: Control Vs severe; P4: Mild Vs moderate; P5: Mild Vs severe; P6: Moderate Vs severe

**(**Table 5) Accuracy of serum TG2 in classification of CSU into mild, moderate and severe subgroups through the determination of sensitivity, specificity, positive predictive value and negative predictive value:

**Table 5:** Sensitivity, specificity, PPV and NPV of serum TG2 in differentiating between mild and moderate and severe cases of CSU

	Differentiation between mild and moderate cases	Differentiation between moderate and severe cases
Cut-off point of serum TG2	> 286.4	> 571.9
AUC	0.809	0.950
Sensitivity	70.00	95.00
Specificity	80.00	90.00
Positive predictive value	77.8	90.5
Negative predictive value	72.7	94.7

Table 6 represents the analysis of the correlation between the serum level of TG2 and the other studied parameters. This analysis is conducted on the level of all cases and each subgroup of CSU patients (mild, moderate and severe). On the level of all cases, the table shows a highly significant positive correlation (direct relationship) between serum level of TG2 and (UAS) as well as with CRP level and negative correlation (inverse relationship) with ESR (2nd hour). For moderate cases of CSU, a significant positive correlation between serum TG2 and lymphocytes values, as well as serum TSH level, is observed. In the group of severe CSU cases, there is a significant negative correlation between age and serum TG2 in addition to the observed significant negative correlation between serum TG2 and TSH levels. There is no significant correlation between serum TG2 and all the other studied parameters illustrated in the table.

**Table 6:** Correlation of serum transglutaminase-2 with other studied parameters all case, mild, moderate and severe cases

	Serum transglutaminase-2							
	All cases		Mild cases		Moderate cases		Severe cases	
	*r*	*P*-value	*r*	*P*-value	*r*	*P*-value	*r*	*P*-value
Age (yrs)	− 0.037	0.780	0.142	0.550	− 0.041	0.862	− 0.449*	**0.047**
UAS	0.814**	**0.000**	–	–	0.041	0.863	0.076	0.749
Hemoglobin	0.190	0.146	0.381	0.098	0.032	0.892	0.146	0.540
Platelets (10^3)	− 0.073	0.579	− 0.168	0.478	− 0.039	0.870	0.015	0.950
TLC	0.016	0.904	− 0.272	0.246	0.290	0.216	0.114	0.631
Neutophils	0.052	0.695	− 0.335	0.149	0.255	0.278	0.193	0.416
Lymphocytes	0.056	0.672	− 0.268	0.254	0.543*	**0.013**	0.128	0.590
Neut/Lymph ratio	0.080	0.545	0.227	0.335	− 0.369	0.109	− 0.100	0.673
Esinophils	− 0.200	0.126	− 0.049	0.836	− 0.352	0.128	− 0.127	0.594
CRP	0.273*	**0.035**	− 0.129	0.587	− 0.136	0.568	− 0.039	0.872
ESR 1st hour	− 0.195	0.136	0.085	0.722	− 0.173	0.466	0.293	0.210
ESR 2nd hour	− 0.267*	**0.039**	0.128	0.591	− 0.088	0.712	0.034	0.888
TSH	− 0.102	0.438	0.080	0.736	0.469*	**0.037**	− 0.485*	**0.030**
Total IgE	− 0.118	0.370	− 0.202	0.394	− 0.250	0.289	− 0.135	0.571

Bold indicates significant statistical values

•*P*-value > 0.05: Non significant; *P*-value < 0.05: Significant; *P*-value < 0.01: Highly significant

Spearman correlation coefficient

Table 7 represents the analysis of the correlation between the serum level of TG2 and the age of onset of the disease in patients with CSU, as well as analysis of the difference in the serum level of TG2 between CSU patients regarding (atopy, blood eosinophils, CRP and serum total IgE). The table shows that there is no significant correlation between serum TG2 and the age of disease onset and non-significant difference in the serum level of TG2 among CSU patients regarding the aforementioned parameters.

**Table 7:** Comparison of serum TG2 between patients with CSU regarding (Presence of atopy, blood eosinophil count, CRP and serum total IgE)

	Serum transglutaminase-2				
	*r*	Category	Median (IQR)	Test	*P*-value
Age of onset (yrs)	0.006	–	–	–	0.966
Presence of atopy	–	Positive	346.3 (211.45–568.45)	1.04	0.297
		Negative	447.95 (267.5–616.7)		
Blood esinophils	− 173	High	346.3 (213.7582.6)	1.25	0.208
		Non-high	447.9 (268.5–767.9)		
CRP	0.216	High	513.55 (272.6–633.1)	0.941	0.347
		Non-high	374.5 (243.1–600.5)		
Total IgE	− 117	High	421.3 (248.4–617.2)	0.527	0.597
		Non-high	433.4 (263.7–746.3)		

•*P*-value > 0.05: Non significant; *P*-value < 0.05: Significant; *P*-value < 0.01: Highly significant

Spearman correlation coefficient

## Discussion

CSU is an immunological disease that is depicted by high prevalence and eminent burden for patients and society. This is attributable to the potent effects posed by the disease on the health-related quality of life that stem from various challenges in disease management. The most prominent is the arbitrary nature of symptoms reinforced by the subjective inconsistent tools for the assessment of activity and severity which reflects on the validity of informed treatment decisions. The results of recent studies have supported the exploitation of blood parameters as potential indicators of the disease activity and severity, yet with inconsistent guidance and indeterminate conclusions concerning the markers currently in clinical application. The identification and validation of a blood marker with coherent association with CSU is vital for the implementation of competent personalized patient-centered treatment plans. This study aimed to determine the relationship between serum levels of TG2 and the severity of the disease in CSU patients. To the best of our knowledge, this is the first study in Egypt to determine the relationship between serum TG2 and severity of CSU.

TG2 is a transamidating acyltransferase protein catalyst that is globally expressed in many cells and tissue types including mast cells. Its biological roles are principally employed through modification of proteins and these entail mechanisms involving cell membrane, extracellular matrix and signal transduction. Remarkably, these functions of TG2 may justify the theories behind its contribution to the development of autoimmune and inflammatory diseases [9]. It participates in the pathogenesis of allergic disorders by means of induction of TH2 allergic response, increase in eicosanoids [10] and boosting IgE production [11]. Accordingly, there is a robust assumption that TG2 is a significant pillar in the pathogenesis of CSU with evolving affirmations for its correlation to severity of the condition.

**Fig. 1:**
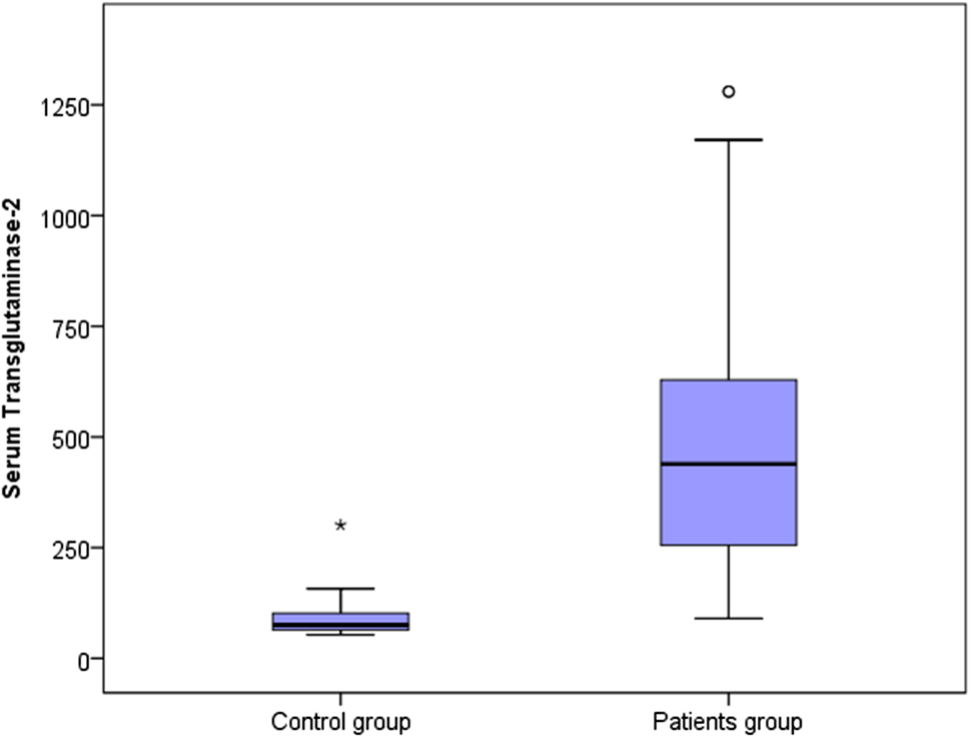
Comparison between control and patients groups regarding serum TG2

Findings from this study revealed elevated serum levels of TG2 in patients with CSU in comparison to the control group as illustrated in Table 3 and Fig. 1 with a cut-off point (> 157.2). Similar findings were reported by a study of Hong et al. [14], who found that the activity of TG2 was significantly increased in the serum samples of patients with CSU compared with the healthy controls. This was also consistent with observations of significantly higher TG2 activity in the sera of CSU patients compared to controls in a previous study of Bae et al. [15]. A former study conducted on murine asthma model demonstrated that TG2 was implicated in the activation of mast cells and hence the production of IgE and release of mediators [16]. A very recent study by Su et al. [17], reported elevated levels of IgE-anti-TG2 in 20% of CSU patients. The significant difference in the levels of serum TG2 between patients with CSU and control group verifies the high potentiality of the relationship between CSU and TG2 and hence imparts more evidence that emphasize the inherent role imposed by TG2 in the pathogenesis of CSU.

**Fig. 2:**
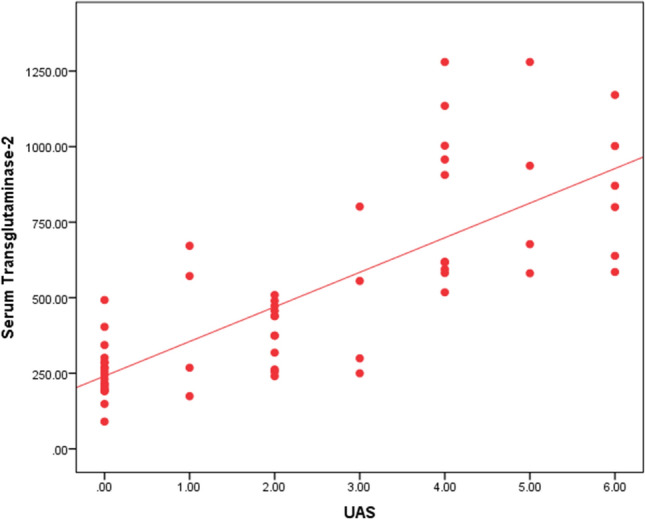
Correlation between serum transglutaminase-2 and UAS among all cases

**Fig. 3:**
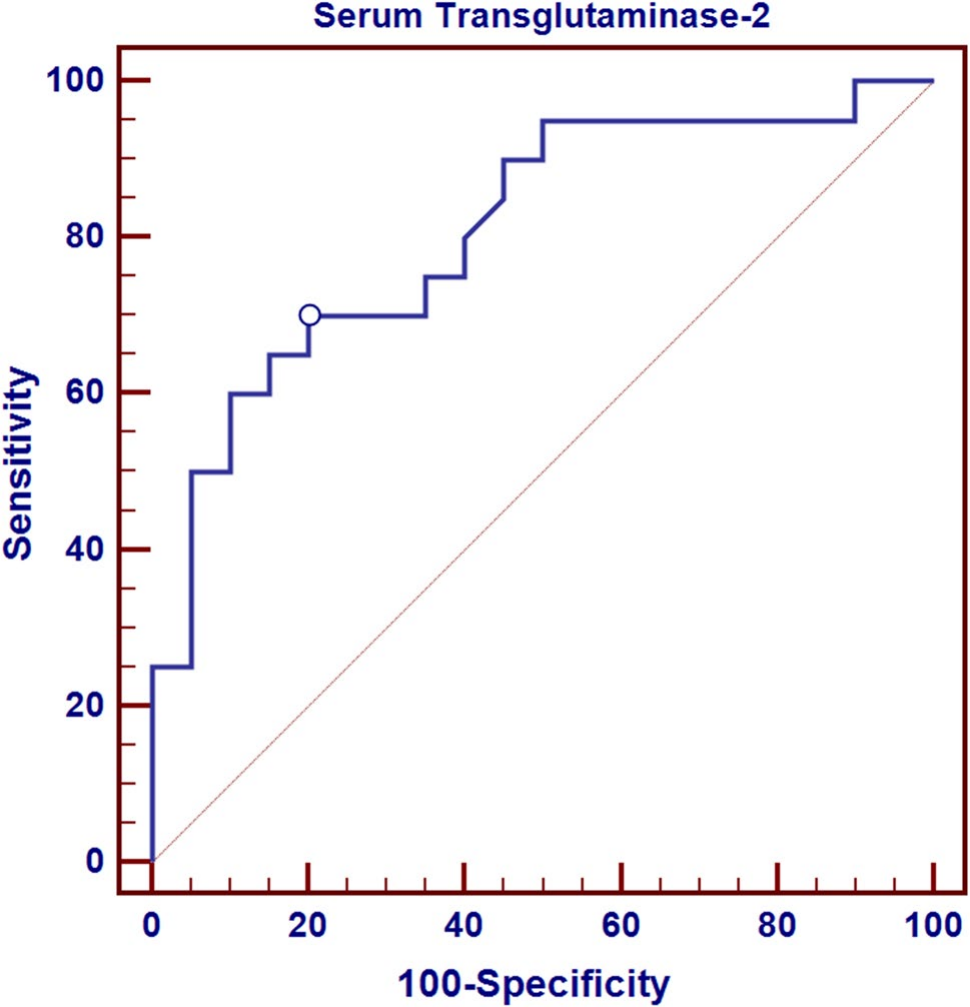
ROC curve for serum TG2 to differentiate between mild and moderate cases

As illustrated in Fig. 2, CSU patients with the highest outcome of (UAS) exhibited the uppermost levels of serum TG2. These results point to the alleged relation between TG2 and the severity of the disease. This is in line with the study of Bae et al. [15] which found significant increase in serum TG2 activity in correlation to disease severity in CSU patients. Conversely, Hong et al. [14] , could not demonstrate correlation between CSU severity and serum levels of TG2. However, this might be explained by the failure to measure UAS at the time of obtaining the serum samples. An alternative explanation could be that, in the concerned study, a remarkable portion of included CSU patients were at the first level of treatment. This rendered the statistical analysis applied according to disease severity incongruous.

**Fig. 4:**
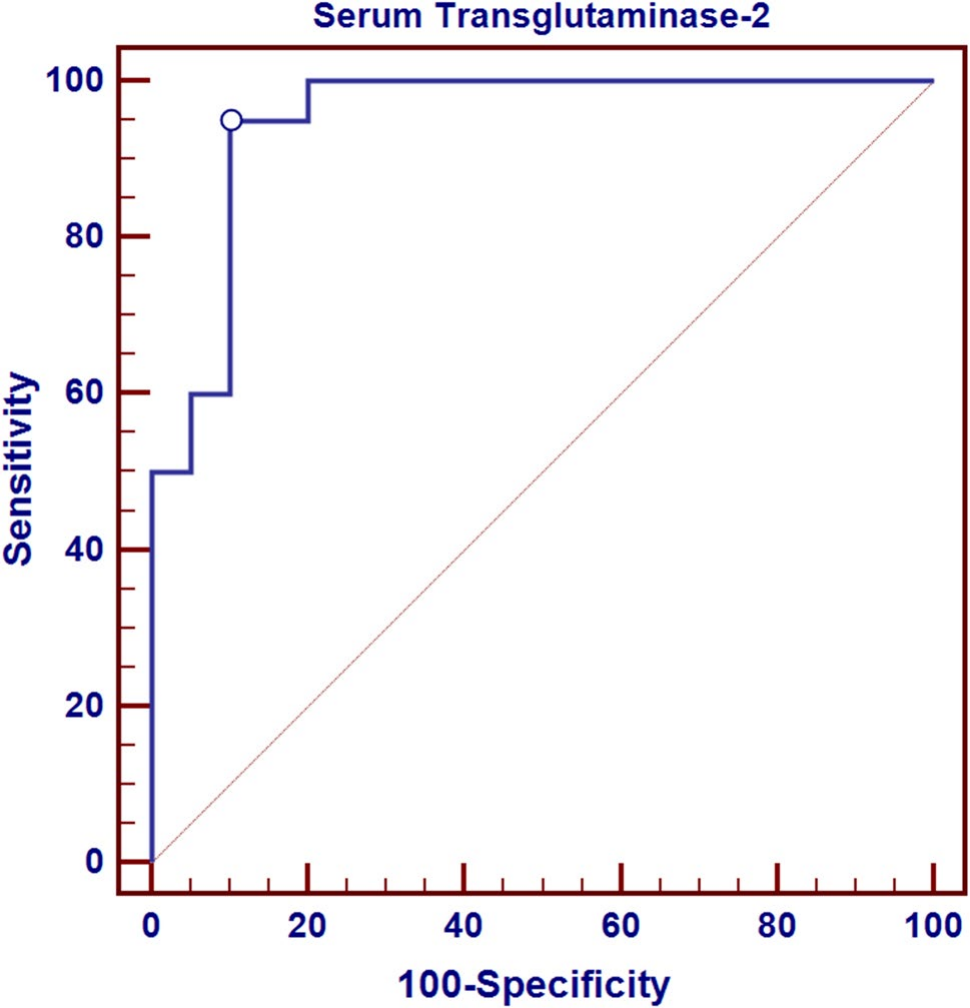
ROC curve for serum TG2 to differentiate between moderate and severe cases

**Fig. 5:**
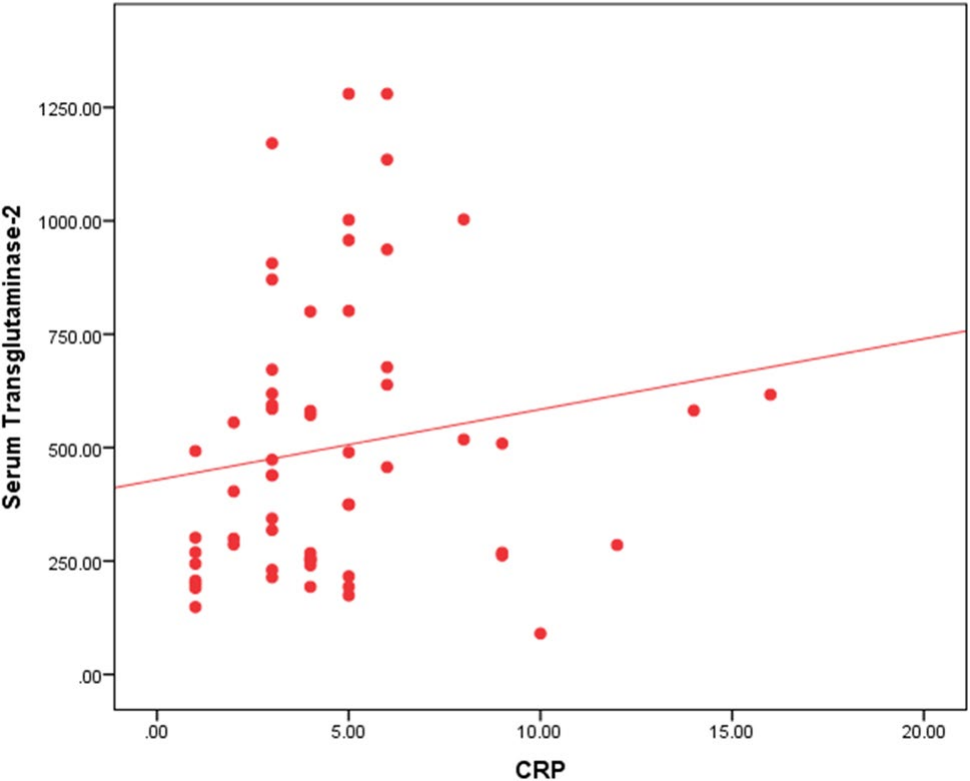
Correlation between serum transglutaminase-2 and CRP among all cases

The present study illustrated that serum TG2 might be beneficial as a parameter to demarcate mild and moderate cases of CSU with a demonstrated cut-off level (as shown in Table 5 and Figs. 3, 4). The pillars of this prospective function were the high sensitivity and specificity (70% and 80% respectively). Moreover, the sensitivity and specificity of serum TG2 to demarcate moderate and severe cases were even higher (95% and 90% respectively). Our findings can be explained by reports from the study of Hong et al. [16], highlighting the part imposed by TG2 in the inflammation process and allergic diseases in regard to allergic asthma where it exerts its role through mediation of IL-33 expression and subsequent Th2 differentiation. Activity of TG2 during mast cell activation upregulates CD40L and boosts production of IgE and this effect is augmented by its increased manifestation in the mast cells of the skin lesions in CSU. The results of our study, in this perspective, were also concordant with Bae et al. [15] who proposed that serum TG2 can be a specific marker of disease activity and severity in CSU leaning on the evidence of the association between TG2 and CSU pathogenesis.

The current study demonstrated a positive correlation between the levels of CRP and CSU activity and severity. The results of our study showed that CRP levels are higher in the group of CSU patients with moderate severity than the mild group and reported the highest values in the group with highest severity. This comes in agreement with previous studies that depicted significant association between CRP levels and CSU activity [18]. Elevated CRP with disease activity can be attributed to the inflammatory response accompanying mast cell activation [19]. However, Criado et al. [20], perceived inconsistent correlation between CRP levels and severity of CSU. Nonetheless, this could be justified by the small size of the study group and the short duration of analysis which were regarded as limitations of the aforementioned study.

The previously highlighted evidence of the correlation between CRP levels and CSU severity support the findings of our study demonstrating a significant association of CRP levels and serum levels of TG2 as illustrated in Fig. 5. CRP is an important parameter that has been involved in different studies concerning the preliminary establishment of beneficial blood biomarkers in CSU. Nevertheless, confronted by the challenge of lack of specificity being incorporated in a vast spectrum of chronic infections and autoimmune diseases, CRP has not been affirmed as a potent conclusive marker in this regard. This might form a pillar for rooting the role of serum TG2 as a marker of disease severity with the competent specificity shown in the present study. Furthermore, setting a correlation analysis between biomarkers paves the way for more comprehensive understanding of CSU pathogenesis and yields a potential for adding a prognostic value.

A more profound and comprehensive understanding of the relationship between serum TG2 and pathogenesis of CSU can be addressed through the implementation of the hypothesis of endotyping of CSU patients. In accordance with the evidence of the salient role of TH2 in CSU pathogenesis, the applied parameters for endotyping are classified into T2 endotype and non T2 endotype markers. T2 endotype elucidates atopic (IgE-mediated) CSU while the non T2 endotype illustrates the non IgE-mediated patterns.

Among the analyzed biomarkers of CSU, the T2 endotyping was defined by the presence of atopy, blood eosinophil count and total IgE which are descriptive of the allergic domain of the pathomechanism. Alternatively, the non T2 endotype was defined by CRP which is a non-specific acute phase reactant. In this perspective, findings of the present study exhibited non significant correlation between age of onset of CSU and serum TG2 as well as non significant difference in serum TG2 among the designated categories (illustrated in Table 7). This is consistent with the study conducted by Bae et al. [15] in which the activity of TG2 was not significantly different according to atopy and portrayed no correlation of serum TG2 with neither blood eosinophil count nor total IgE [15]. To the best of our knowledge, no studies till the current time have displayed evidence against these findings.

Further research should investigate the relationship between serum TG2 and severity of disease in patients with CSU. This could be done by conducting a larger-scale study with a more diverse population and in more than one center. Moreover, more solid information could be granted through the provision of a follow-up assessment of serum TG2 level after at least 6 months of treatment.

In conclusion, the findings of our work indicated that serum TG2 may have a pivotal role as a marker of severity in patients with CSU with the benefit of retaining convenient sensitivity and specificity in outlining mild, moderate and severe subpopulations.

## Abbreviations

ANA: Antinuclear antibody
AUC: Area under the curve
CBC: Complete blood count
CIs: Confidence intervals
CRP: C-reactive protein
CSU: Chronic spontaneous urticaria
CUQ2oL: Chronic urticaria quality of life
ESR: Erythrocyte sedimentation rate
IL-6: Interleukin-6
IL-33: Interleukin-33
MCs: Mast cells
NPV: Negative predictive value
PLA2: Phospholipase A2
PPV: Positive predictive value
RPM: Revolutions per minute
TG2: Transglutaminase-2
TH2: T-helper 2
TLC: Total leukocyte count
TSH: Thyroid-stimulating hormone
UAS: Urticaria activity score
UCT: Urticaria control test
